# Simultaneous generation of high-efficiency broadband asymmetric anomalous refraction and reflection waves with few-layer anisotropic metasurface

**DOI:** 10.1038/srep35485

**Published:** 2016-10-20

**Authors:** Zhancheng Li, Wenwei Liu, Hua Cheng, Jieying Liu, Shuqi Chen, Jianguo Tian

**Affiliations:** 1 The Key Laboratory of Weak Light Nonlinear Photonics, Ministry of Education, School of Physics and TEDA Institute of Applied Physics, Nankai University, Tianjin 300071, China

## Abstract

Optical metasurfaces consisting of single-layer nanostructures have immensely promising applications in wavefront control because they can be used to arbitrarily manipulate wave phase, and polarization. However, anomalous refraction and reflection waves have not yet been simultaneously and asymmetrically generated, and the limited efficiency and bandwidth of pre-existing single-layer metasurfaces hinder their practical applications. Here, a few-layer anisotropic metasurface is presented for simultaneously generating high-efficiency broadband asymmetric anomalous refraction and reflection waves. Moreover, the normal transmission and reflection waves are low and the anomalous waves are the predominant ones, which is quite beneficial for practical applications such as beam deflectors. Our work provides an effective method of enhancing the performance of anomalous wave generation, and the asymmetric performance of the proposed metasurface shows endless possibilities in wavefront control for nanophotonics device design and optical communication applications.

Metasurfaces are periodic single-layer artificial nanostructure arrays with sub-wavelength unit-cells and thicknesses, which can overcome the physical limitations imposed by natural materials and provide exceptional capabilities for manipulating waves with greater precision^1^,^2^,^3^,^4^. Optical metasurfaces have recently attracted a great deal of attention since the freedom they provide in controlling wavefront offers intriguing possibilities in the field of nanophotonics. A series of exotic applications and associated optical devices including anomalous refraction and reflection^5^,^6^,^7^,^8^,^9^,^10^, ultrathin flat lenses^11^,^12^, vortex beam generation^13^,^14^,^15^, the spin-Hall effect of waves^16^,^17^, holograms^18^,^19^,^20^,^21^,^22^,^23^, and polarization management^24^,^25^ have been proposed and exploited using metasurfaces. Although such great achievements have been made by using low-loss single-layer metasurfaces and simple fabrication techniques, the limited interaction between waves and single-layer metasurfaces has induced inherent defects in the efficiency and bandwidth of single-layer metasurface-based optical devices^3^,^4^,^5^,^6^,^7^,^8^, resulting in limited wave manipulation controllability and preventing such devices from being used in practical applications.

Recent advances in few-layer metasurfaces provide an alternative method of overcoming the drawbacks of single-layer metasurfaces. Grady *et al*. proposed a broadband near-perfect anomalous refraction wave generated by a three-layer metasurface in the THz range^26^. Pfeiffer *et al*. produced a high-performance metasurface lens that both focused light and controlled its polarization with four cascaded metasurfaces in the near-infrared range^27^. Li *et al*. proposed a dual-layer plasmonic metasurface to simultaneously manipulate the phase and polarization of the transmitted light and obtain an arbitrary spatial field distribution of the optical phase and polarization direction^28^. Few-layer metasurfaces designed with near-field wave interference and interlayer resonance have improved the efficiency and controllability of wave manipulation and have thus provided novel functionality and more degrees of freedom to manipulate the propagation, polarization, and phase amplitude of light^29^,^30^.

Harnessing light for modern nanophotonics applications often involves the control and manipulation of wavefront. The fundamental purpose of wavefront-control applications is to achieve the anomalous refraction and reflection of light. Although previous approaches in few-layer metasurfaces have dramatically enhanced the efficiency of anomalous light, high-efficiency broad-bandwidth anomalous refraction and reflection waves still have not been effectively generated simultaneously. Moreover, an alternative method of improving the intensity of anomalous waves while simultaneously suppressing that of normal ones is also needed so that few-layer-metasurface-based wavefront controls can be used in a wide range of applications.

Here, we propose an anisotropic metasurface to simultaneously generate broadband high-efficiency asymmetric anomalous refraction and reflection waves for circularly polarized incident waves in the near-infrared range. The waves are theoretically predicted and demonstrated using simulation. More importantly, the proposed metasurface not only improve the efficiency and bandwidth of the generated anomalous waves but also suppress the normal reflection and transmission waves in a broad bandwidth, thereby overcoming the main defect in most previous works. More specifically, the proposed metasurface can split an arbitrarily polarized incident beam into two anomalous waves with same polarization state propagating in opposite directions and the polarization states of anomalous waves are orthogonally for opposite incident directions, which provides a powerful method of designing optics systems in nanophotonics.

## Theoretical Analysis

The introduction of polarization conversion and the continuous phase gradients generated by metasurfaces usually contribute to the generation of anomalous waves. Following the approach previously discussed^4^, equal polarization conversion amplitudes and the corresponding 2*π* continuous phase gradient along the direction perpendicular to the wave propagation are necessary for anomalous wave generation. When the orientation angle of the metasurface with circular polarization conversion changes *θ*, the phase of the cross polarized wave will change ±*2θ* for LCP and RCP incident lights, respectively^8^,^12^. Thus, a 0–2*π* continuous phase gradient of circular polarized conversion waves can be achieved in metasurfaces by rotating the array of the unit-cell structures along the geometric axis parallel to the wave propagation direction from 0 to *π*. The relation between the incident angle *θ*_*i*_, and the anomalous refraction angle *θ*_*t*_, can then be obtained by the generalized Snell’s law^5^,^8^,^9^:


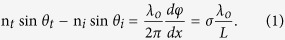


Similarly, for the anomalous reflection angle *θ*_*r*_,


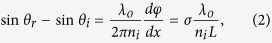


where *dφ*/*dx* indicates a suitable phase gradient along the metasurface, and *λ*_*o*_ represents the wavelength in free space. *L* represents the periodic length of the metasurface array for the 2*π* continuous phase gradient. The phase gradient *dφ*/*dx* of the cross polarized wave in Eqs (1) and (2) are opposite for LCP and RCP cross polarized waves with positive and negative signs, respectively. *σ* = ±1 indicating the sign of the phase gradient corresponds to the helicity of left-handed circular polarization (LCP) and right-handed circular polarization (RCP) incident waves propagating along −*z* direction.

Thus, the efficiency of the anomalous wave generation is mainly decided by the equal polarization conversion efficiency of the unit-cell structures. We consider the incoming plane waves propagating along the forward (+*z*) and backward (−*z*) directions, with the electric fields as^31^,^32^,^33^


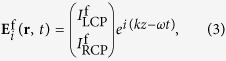



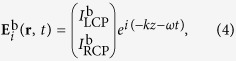


where *ω, k, I*_LCP_, and *I*_RCP_ represent the frequency, wave vector, and complex amplitudes, respectively, and the superscripts “f” and “b” indicate the forward and backward directions. The outgoing fields for two opposite directions is then given by:


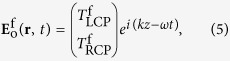



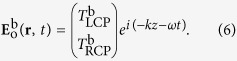


The Scattering matrix **S**, then relates the four complex amplitudes as follows^34^:


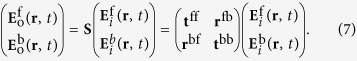


In more detail,


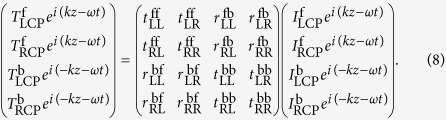


**Figure 1:**
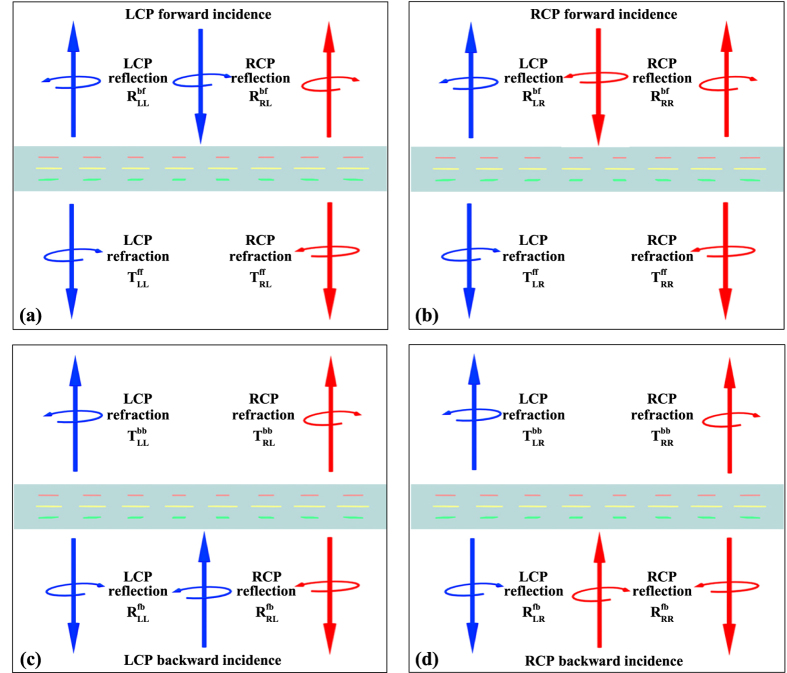
A diagram of S matrix elements. (**a**) Elements related to LCP forward incidence. (**b**) Elements related to RCP forward incidence. (**c**) Elements related to LCP backward incidence. (**d**) Elements related to RCP forward incidence.

The subscript “ij” of the **S** matrix elements indicates the polarization state is transformed from “j” to “i”, and the superscript “kl” indicates the propagation direction from “l” to “k”, as shown in Fig. 1. Previous approach for anomalous wave generation of circular polarized waves in metasurfaces always involves nanorod unit cells, which is mirror-symmetric with respect to a plane parallel to the *z* axis^9^,^12^,^14^. For this kind of structure, the relationship between transmission coefficients of **S** matrix are 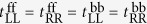 and 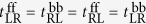. The relationship between reflection coefficients of **S** matrix are 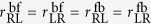 and 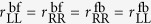. Thus, the **S** matrix can be simplified to^31^:


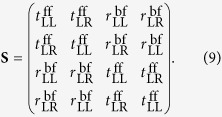


Thus, the circular polarization conversion efficiencies for LCP and RCP incident waves are identical because the amplitudes of the corresponding **S** matrix elements are the same. Anomalous refraction and reflection waves have previously been generated by designing high polarization conversion coefficients 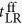 and 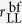, respectively. Because of the limited interaction between incident waves and single-layer metasurfaces, the amplitudes of 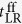 and 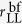 are low and for few-layer metasurfaces, 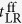 and 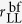 cannot simultaneously reach acceptable values. Furthermore, 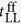 and 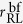, corresponding to the normal refraction and reflection waves, respectively, previously existed and hindered the practical application of few-layer metasurfaces. Thus, high-performance anomalous reflection and refraction waves still have not been simultaneously generated. More specifically, the **S** matrix elements for oppositely propagating incident waves (*i*.*e*., propagating in the forward and backward directions) are identical, signifying that the nanorod-based metasurfaces is uniform for incident waves propagating in opposite directions.

For reciprocal structure with mirror symmetry perpendicular to the *z*-axis and at most a C_2_ symmetry with respect to the z axis, the relationship between transmission coefficients of **S** matrix are 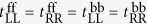, 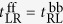 and 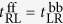. The relationship between reflection coefficients of **S** matrix are 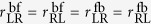, 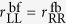 and 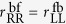. Then, the **S** matrix of this kind of structure can be simplified to^31^,^32^:


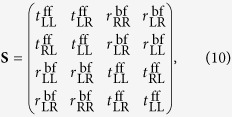


where the coefficients 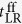 and 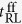 (or 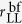 and 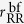) are not identical, and both 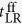 and 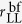 (or 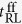 and 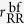) can attain high values while the other elements are close to zero because of the few-layer anisotropic design of the metasurface. This characteristic of the **S** matrix for the reciprocal anisotropic few-layer metasurface indicate that asymmetric circularly polarized anomalous refraction and reflection waves can be simultaneously generated while normal reflection and refraction waves are simultaneously suppressed. Thus, the ideal **S** matrix for a few-layer anisotropic metasurface simultaneously generating asymmetric anomalous reflecting and refracting waves can be simplified to


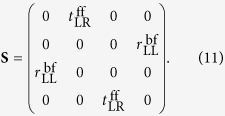


## Results and Discussion

**Figure 2:**
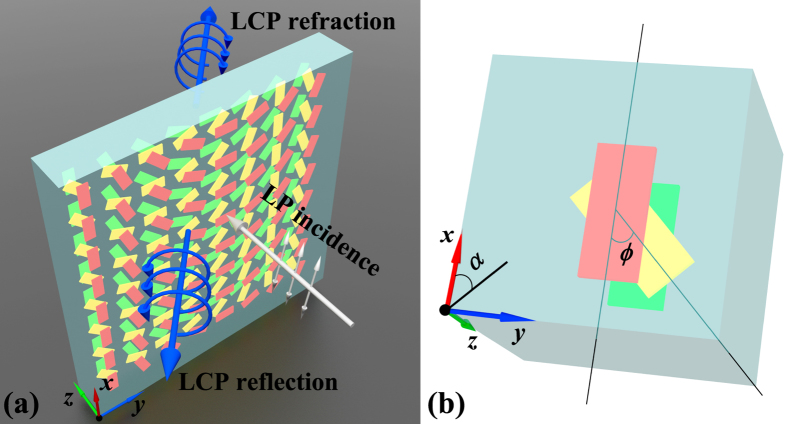
Schematic of designed reciprocal anisotropic metasurface. (**a**) An artistic rendering of asymmetric anomalous wave generation for linear polarized forward incident wave. Metasurface array consisting of eight basic unit cells designed with same geometry and step-by-step rotation angle of −*π*/8 along +*y* direction to generate constant phase gradient. (**b**) Detailed geometry of unit cell. Angle *α* between upper nanorod and *x*-axis indicates orientation angle.

A three-layer anisotropic metasurface array with a mirror symmetry perpendicular to the *z*-axis and a C_2_ symmetry with respect to the *z* axis is designed to approximately fit the ideal **S** matrix and simultaneously generate high-performance asymmetric anomalous reflection and refraction waves. The array, showed in Fig. 2(a), consists of 8 basic unit cells realized with the same geometry but linearly varied orientations with a stepwise rotation of −*π*/8 along the +*y* direction. Figure 2(b) shows the detailed geometry of the unit cell. Three 530-nm-long, 230-nm-wide, 30-nm-thick gold nanorods are embedded into the SiO_2_ substrate. The upper (red) and lower (green) nanorods are parallel to the *x*-axis, and the angle *ϕ*, between the upper nanorod and the middle one (yellow) is 45°. The distance between the nanorods and the thickness of the SiO_2_ covering on the upper nanorod are both *d* = 250 nm. The periods of the unit cells are *P* = 800 nm along the *x* and *y* directions; thus, the periodicity of the metasurface array is 800 and 6400 nm in the *x* and *y* directions, respectively. Numerical simulations are conducted using Computer Simulation Technology MICROWAVE STUDIO (CST MWS) to analyze the characteristics of the proposed metasurfaces^35^,^36^.

**Figure 3:**
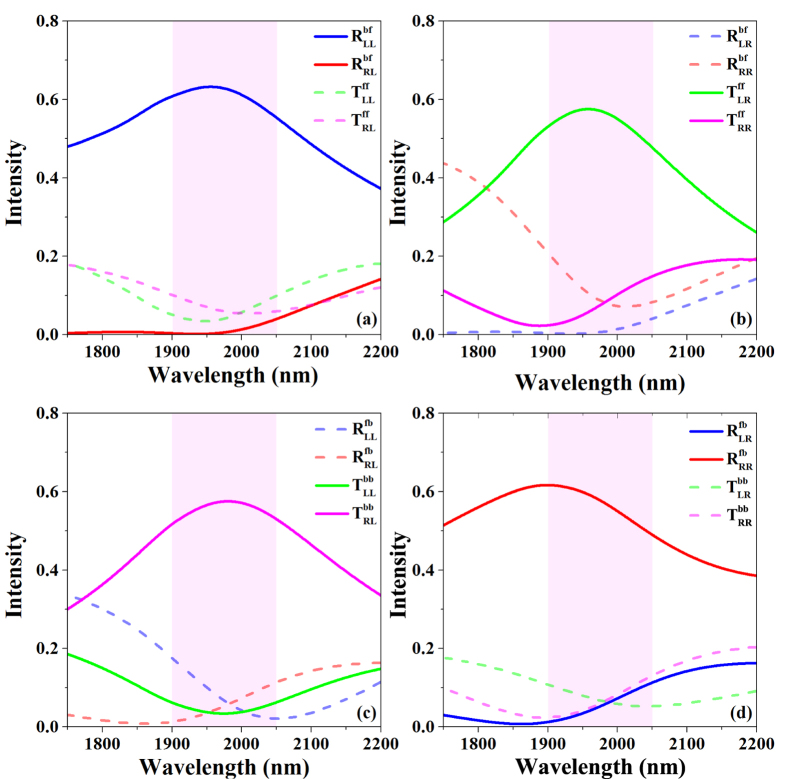
Simulated results for squared moduli 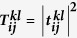 and 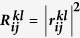 of S matrix elements of unit cell structure. (**a**,**c**) LCP and (**b**,**d**) RCP incident waves propagated along forward and backward directions, respectively. Shadow areas indicate the waveband, where the squared moduli of 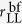, 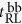, 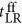, and 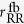 are more than 45% while the squared moduli of other elements are no more than 20%.

Because the phase conditions for asymmetric anomalous refraction and reflection waves can be easily satisfied by rotating the unit cell structure, the polarization conversion amplitude is considered first. Rotating the basic unit cell (as shown in Fig. 2(b)) does not affect the amplitudes of the elements in the **S** matrix; thus, the **S** matrix of the basic unit cell is investigated and optimized to approach the ideal amplitude conditions (*i*.*e*., the ideal **S** matrix, as shown in Eq. (11)). Figure 3 shows the simulated results for the squared moduli 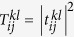 and 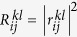 of the **S** matrix for the unit cell, where the LCP and RCP incident waves propagate from the forward and backward directions, respectively. The shadow areas indicate the waveband from 1900 to 2050 nm where the **S** matrix approximately fits the ideal one in Eq. (11). As showed in Fig. 3(a), 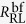, 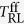, and 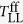 are close to zero while 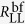 is considerably large for the LCP normal incident wave propagating from the forward direction. Accordingly, only the RCP refraction wave is generated from the LCP normal incident wave propagating from the backward direction, as showed in Fig. 3(c). For the RCP normal incident wave propagating from the forward direction, 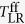 is several times larger than 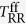, 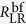, and 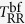 while 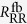 is several times larger than 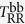, 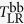, and 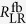 for the RCP normal incident wave propagating from the backward direction, as showed in Fig. 3(b,d). The amplitudes of the proposed **S** matrix elements 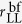, 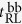, 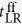, and 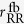 seem to be several times larger than the other elements in the shadow areas. Thus, the other elements whose amplitude is negligible are treated as 0. More specifically, the relation between the **S** matrix transmission elements described by Eq. (10) is verified in Fig. 3. The difference between the reflection and refraction elements is due to the difference between the refractive indexes of air and the substrate, indicating that the unit cell is not strictly symmetric. However, such small differences only have a negligible effect on whether the amplitude conditions are satisfied. The relationship between **S** matrix elements in our proposed unit cell of metasurface is attributed to the anisotropic few-layer structure design with a mirror symmetry perpendicular to the *z*-axis and a C_2_ symmetry with respect to the *z* axis. While, the high efficiency is attributed to the near-field wave interference and interlayer resonance in few-layer structure^29^,^31^. In addition, it is worth mentioned that the primary loss in our designed few-layer metasurface is attributed to the enhanced absorption, which is also associated with the interference and the near-field coupling between layers.

**Figure 4:**
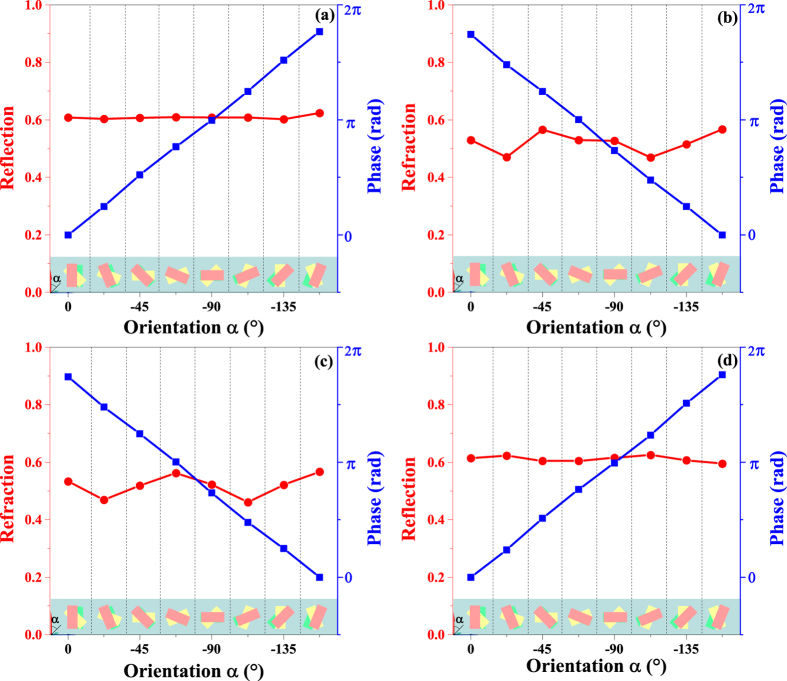
Simulated results for refraction intensity, reflection intensity, and phase shift along metasurface array. For (**a**,**c**) LCP and (**b**,**d**) RCP incident waves propagated along forward and backward directions, respectively. Wavelength is fixed at 1900 nm.

We next consider the amplitude and phase conditions of the metasurface array for anomalous wave generation. A suitable constant gradient of phase discontinuity is achieved by the metasurface array consisting of 8 basic unit cells designed with the same geometry but linearly varied orientations with a stepwise rotation of −*π*/8 along the +*y* direction (as shown in Fig. 2(a)). Figure 4 shows the simulated results for the refraction intensity, reflection intensity, and phase shift along the metasurface array for 1900 nm LCP and RCP normal incident waves propagating along the forward and backward directions, respectively. For the LCP forward-propagating normal incident wave, the basic unit cell generate only LCP reflection wave at >60% intensity, as showed in Fig. 4(a). The corresponding phase gradient along the metasurface array vary from 0 to 2*π* when the orientation angle *α*, is varied from 0 to *π*, which is consistent with the theoretical prediction. For the LCP backward-propagating normal incident wave, the basic unit cell generate only RCP refraction wave at ~50% intensity and a phase gradient from 0 to 2*π*, as showed in Fig. 4(c). If the effect of the difference between the refractive indexes of air and the SiO_2_ substrate is ignored, the metasurface array can also be treated as a symmetric anisotropic system, meaning it reverses its handedness for circularly polarized incident waves propagating from opposite sides. Thus, the anomalous waves generate from the RCP normal incident wave are opposite to those generated from the LCP normal incident wave (as indicated by Eq. (11)), which can be easily verified in Fig. 4(b,d). Thus, the proposed metasurface array simultaneously satisfies the phase and amplitude conditions for simultaneously generating asymmetric anomalous refraction and reflection waves.

**Figure 5:**
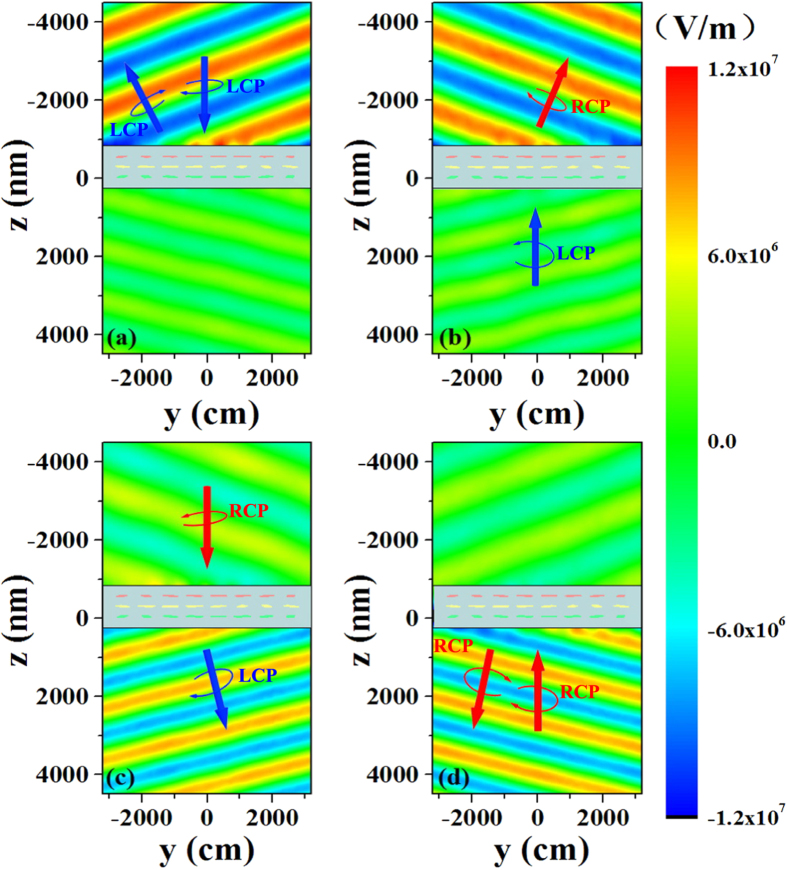
Simulated results for anomalous refraction and reflection waves generated by reciprocal anisotropic metasurface array. A time snapshot of the amplitude of the electric field for (**a**,**b**) LCP and (**c**,**d**) RCP normal incident waves propagating along forward and backward directions, respectively. Wavelength is fixed at 1900 nm.

**Figure 6:**
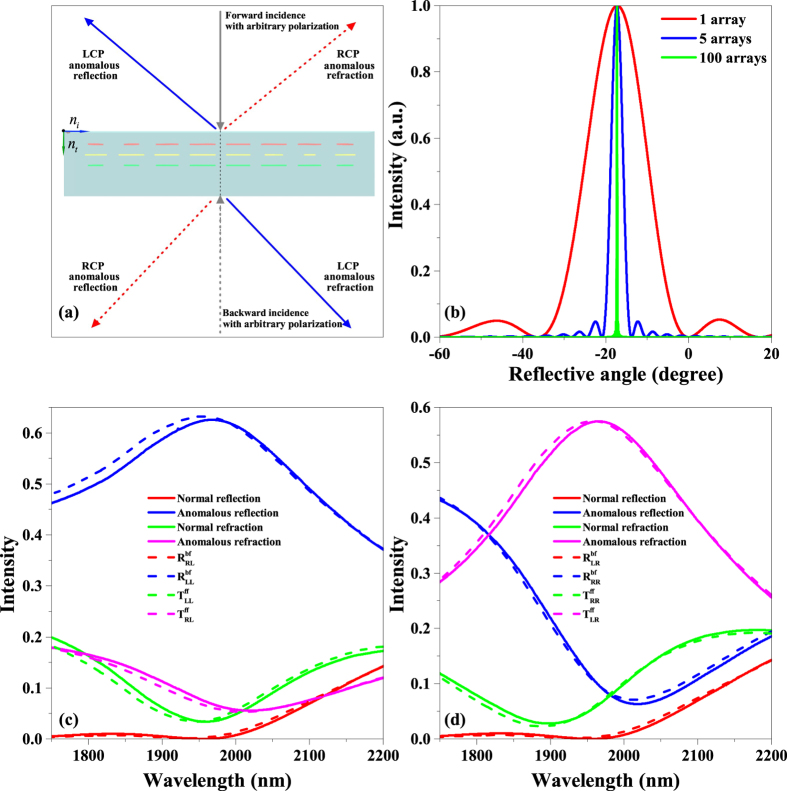
Theoretical illustration and simulated efficiency results of asymmetric anomalous refraction and reflection waves generated. (**a**) Theoretical illustration of asymmetric anomalous waves generated. Using arbitrarily polarized incident wave propagating from either forward (solid line) or backward (dotted line) direction. (**b**) Calculated diffraction pattern of LCP anomalous reflection for different numbers of array along *y* direction generated by LCP forward normal incidence. (**c**,**d**) Comparison between the intensity of the normal and anomalous waves of metasurface, and the squared moduli of the relevant **S** matrix elements of a unit cell for LCP and RCP forward normal incidence, respectively.

The distribution of the electric fields for the anomalous waves is simulated for 1900 nm LCP and RCP normal incident waves propagating along the forward and backward directions, respectively, to intuitively show the asymmetric anomalous refraction and reflection waves. The simulated time snapshot results are showed in Fig. 5. For the LCP normal incident wave propagating along the forward direction (showed in Fig. 5(a)), the intensity of the anomalous refraction wave is almost several times smaller than that of the anomalous reflection wave, meaning that only the LCP anomalous reflection wave are generated. For the LCP normal incident wave propagating along the backward direction, only the RCP anomalous refraction wave are generated, as showed in Fig. 5(b). For the RCP normal incident waves propagating from opposite directions, the simulated results showed in Fig. 5(c,d) are the opposite of those obtained for the LCP normal incident waves. The simulated results are consistent with the theoretical predictions and confirm that the proposed metasurface array approximately generates the desired anomalous refraction and reflection waves. Moreover, because arbitrarily polarized incident waves can be decomposed into LCP and RCP components, such waves propagating from the forward or backward direction can simultaneously generate anomalous refraction and reflection waves with same polarization state and the polarization state of generated anomalous waves are orthogonally for these two opposite incident directions, as indicated by Fig. 6(a). This asymmetric anomalous wave generation provides a new degree of freedom for wavefront control and deflection.

**Figure 7:**
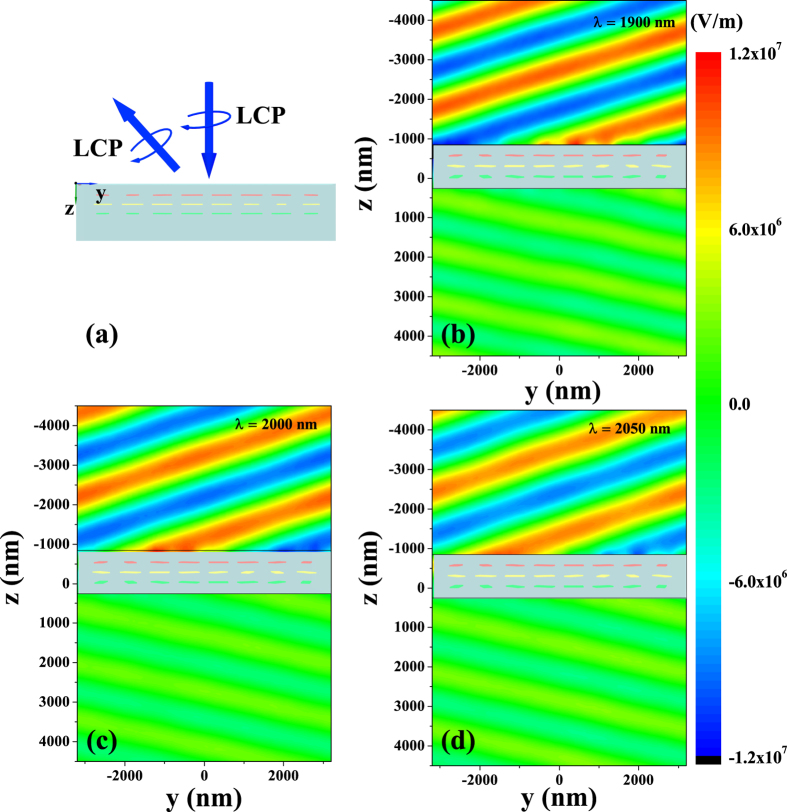
Simulated results for anomalous reflection generated by reciprocal anisotropic metasurface array with LCP normal incident wave propagating along forward direction. (**a**) Schematic of anomalous reflection of LCP normal incident wave propagating along forward direction. A time snapshot of the amplitude of the electric field to show the anomalous reflection generation with wavelength fixed at (**b**) 1900, (**c**) 2000, and (**d**) 2050 nm.

The efficiency or intensity of the anomalous refraction and reflection waves relates to the squared moduli of the relevant **S** matrix elements of a unit cell. As the periodicity of the metasurface array in *y* direction is longer than the wavelength in the designed effective bandwidth, the number of array of the proposed metasurface will affect the diffraction pattern^37^. We calculated the diffraction pattern of LCP anomalous reflection for different numbers of array along *y* direction generated by LCP forward normal incidence in Fig. 6(b). To analyze the efficiency of the beam refraction and reflection in proposed metasurface, we calculated the intensity of the normal waves and the anomalous waves of the metasurface with infinite array, and also the squared moduli of the relevant **S** matrix elements of a unit cell for LCP and RCP forward normal incidences, as shown in Fig. 6(c,d). The high order diffractions are not given as the intensity is close to zero. Results show that the efficiency of metasurface is in consistent with the squared moduli of the relevant **S** matrix elements of a unit cell. The small differences between the intensity of the normal and anomalous waves of metasurface, and the squared moduli of the relevant **S** matrix elements of a unit cell are mainly attributed to the difference of the squared moduli of the relevant **S** matrix elements in each unit cell of an array. Corresponding results for normal backward incidence are in good agreement with the forward one because the proposed metasurface is mirror symmetry perpendicular to the *z*-axis. It is worth mentioning that the normal refraction and reflection are close to zero around the 1900 nm wavelength. Thus, the normal transmission or reflection is low and the anomalous waves are the predominant ones. The suppression of the normal transmission and reflection is well useful to the further research of metasurface. Furthermore, the proposed metasurface maintains high efficiency in a broad bandwidth. We simulated the broadband performances of the proposed metasurface using 1900, 2000, and 2050 nm LCP normal incident waves propagating along the forward direction, as shown in Fig. 7. The anomalous refraction waves generated by the 2000 and 2050 nm incident waves are consistent with those generated by the 1900 nm one. The simulated results in Figs 6 and 7 show that the asymmetric anomalous waves can be realized from 1900 to 2050 nm. Thus, the proposed metasurface array can simultaneously generate broadband high-efficiency anomalous refraction and reflection waves. The efficiency of our proposed metasurface is higher than 45% in a 150 nm bandwidth and the intensity of normal waves are no more than 20%. With this criterion, the bandwidths of typical single-layer metasurface are equal to zero^5^,^6^,^7^,^8^, thereby the proposed few-layer design overcomes the limited bandwidth and low efficiency of previous single-layer devices and is proved to be quite beneficial for practical applications.

## Conclusions

In conclusion, we have proposed a few-layer metasurface to simultaneously generate high-efficiency broadband asymmetric anomalous refraction and reflection waves in the near-infrared range. On the basis of the results of the theoretical analysis, a few-layer anisotropic metasurface is designed, optimized, and used to simulate the generation of asymmetric anomalous refraction and reflection waves. The simulation results are consistent with the theoretical prediction, and high-efficiency broadband asymmetric anomalous refraction and reflection waves are generated. Arbitrarily polarized incident waves propagating from either forward or backward directions can be split into two anomalous waves propagating in opposite directions with the same polarization state. Moreover, the polarization state of generated anomalous waves are orthogonally for these two opposite incident directions. This characteristic is quite useful for beam splitting, polarization selection, optical communication and other applications based on the generation of anomalous beams with designated polarization state and propagation direction.

## Methods

Numerical simulations were carried out with the use of Computer Simulation Technology MICROWAVE STUDIO (CST MWS). In our simulations, the unit cell boundary conditions were set in the *x* and *y* directions representing a periodical structure, and an open (perfectly matching layer) boundary was defined in the *z* direction for the light incidence and transmission while the excitation source was either a left- or a right-handed circularly polarized plane wave. The permittivity of the SiO_2_ was taken as 2.25, and the dielectric constant data for gold was directly applied from the Handbook of Optical Constants of Solids^36^. Moreover, the permittivity of the gold in our simulation can be expressed with Drude mode 
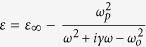
, where *ε*_∞_ = 1, *ω*_*p*_ = 1.59 × 10^15^ *s*^−1^, *γ* = 1.94 × 10^13^ *s*^−1^ and *ω*_0_ = 6.85 × 10^13^ *s*^−1^. A single-layer nanorod was firstly designed and simulated to obtain a resonance at the near-infrared regime. Then, a three-layer structure was designed to form an anisotropic structure with **S** matrix as Eq. (10) predicted. After that, the efficiency and bandwidth of the proposed three-layer structure were optimized to make the **S** matrix of the structure approach the ideal one (as indicated in Eq. (11)) by manipulation of the distance between each layer and fine adjustment of the nanorod structure parameters.

## Additional Information

**How to cite this article**: Li, Z. *et al*. Simultaneous generation of high-efficiency broadband asymmetric anomalous refraction and reflection waves with few-layer anisotropic metasurface. *Sci. Rep.* **6**, 35485; doi: 10.1038/srep35485 (2016).
